# Density and Stability of Soil Organic Carbon beneath Impervious Surfaces in Urban Areas

**DOI:** 10.1371/journal.pone.0109380

**Published:** 2014-10-09

**Authors:** Zongqiang Wei, Shaohua Wu, Xiao Yan, Shenglu Zhou

**Affiliations:** 1 School of Geographic and Oceanographic Science, Nanjing University, Nanjing, China; 2 School of Environmental and Land Resource Management, Jiangxi Agricultural University, Nanchang, China; Tennessee State University, United States of America

## Abstract

Installation of impervious surfaces in urban areas has attracted increasing attention due to its potential hazard to urban ecosystems. Urban soils are suggested to have robust carbon (C) sequestration capacity; however, the C stocks and dynamics in the soils covered by impervious surfaces that dominate urban areas are still not well characterized. We compared soil organic C (SOC) densities and their stabilities under impervious surface, determined by a 28-d incubation experiment, with those in open areas in Yixing City, China. The SOC density (0–20 cm) under impervious surfaces was, on average, 68% lower than that in open areas. Furthermore, there was a significantly (*P*<0.05) positive correlation between the densities of SOC and total nitrogen (N) in the open soils, whereas the correlation was not apparent for the impervious-covered soils, suggesting that the artificial soil sealing in urban areas decoupled the cycle of C and N. Cumulative CO_2_-C evolved during the 28-d incubation was lower from the impervious-covered soils than from the open soils, and agreed well with a first-order decay model (*C*_t_ = *C*_1_+*C*_0_(1-*e* ^-kt^)). The model results indicated that the SOC underlying capped surfaces had weaker decomposability and lower turnover rate. Our results confirm the unique character of urban SOC, especially that beneath impervious surface, and suggest that scientific and management views on regional SOC assessment may need to consider the role of urban carbon stocks.

## Introduction

At present more than half of the world's population resides in cities and towns, and the percentage of urban population is projected to increase to 70% by 2050 [1]. As a result, urban areas are increasing in extent at a greater pace than any other land use type [2]. The rapid expanding of urban areas have caused large areas of agricultural, pasture, or forest soils to be changed to urban soils [3], [4]. To date, urban land is estimated to cover 9% of the continent [5], in which the impervious surface (e.g., buildings, roads, and other pavements) is estimated to cover nearly 580 000 km^2^ globally, an area larger than France [6]. Presently, the resulting impacts of the impervious surfaces on urban soil function, including soil organic carbon (SOC) storage and dynamics, remain largely unknown.

Yet a growing body of literature suggest that urban soils still have robust C storage capacity [7]–[10], especially in the areas covered by green vegetation (e.g., meadow, forest) which could provide fairly C sequestration [11], [12]. Generally, the SOC storage capacity of urban open soils (without impervious surfaces) is comparable to that of adjacent agricultural soils and varies highly amongst different cities/regions, which is might be controlled by several factors, such as urbanization histories, land use types, soil parent materials, topography, and climate (Table 1). However, the storage and turnover of SOC beneath impervious surfaces are still poorly characterized due to their inaccessibility. The impervious surface in urban area is still rapidly expanding due to urbanization, thus it is critical to investigate the SOC stocks and dynamics beneath impervious, to provide accurate inventories in estimates of the entire SOC storage in urban areas, and to promote our understanding of the net impact of urbanization on terrestrial C pools. The relatively limited previous studies suggest that, in urban areas, the SOC density, an important parameter to calculate SOC storage, is significantly lower under impervious surfaces than in open sites [8], [15]. These studies mainly focus on the amount or stocks of SOC in urban areas, whereas few attempts are made to study the SOC dynamics in the impervious-covered soils.

**Table 1:** Soil organic carbon densities (kg m^−2^ at a depth where indicated) for urban soils located in different cities.

City	Latitude/longitude	Urban SOC density	Suburban SOC density	Adjacent agricultural SOC density	Literature cited
		kg m^−2^			
Liverpool, UK	53.4° N/2.98° W	4^a^	–	–	[13]
Boston, U.S.A.	42.35° N/71.06° W	4.02–4.24^b^	3.33–3.99	2.83–4.29	[14]
New York, U.S.A.	42.34° N/75.19° W	5.67^a^	–	–	[15]
New York, U.S.A.	42.34° N/75.19° W	5.1^c^	3.5	3.4	[7]
Baltimore, U.S.A.	39.28° N/76.62° W	4–7^a^	–	–	[7]
Chuncheon, South Korea	37.87° N/127.73° E	2.48^d^	–	3.16	[16]
Phoenix, U.S.A.	33.45° N/112.06° W	0.5–1.1^b^	–	0.75	[17]
Nanjing, China	32.05° N/118.77° E	4.52^e^	–	–	[18]
Chongqing, China	29.56° N/106.57° E	2–3.6^e^	–	–	[19]

a, 0–15 cm; b, 0–10 cm; c, 0–100 cm; d, 0–60 cm; e, 0–20 cm; –, not determined.

Cities were ranked by latitude.

Here we collected some urban soil samples from typical impervious-covered and open areas in Yixing City, China, an area experienced rapid urbanization these decades. The main objectives of this study were to investigate the SOC density underlying impervious surfaces in urban areas, and to further study the stability or decomposability of SOC in the sealing environment.

## Materials and Methods

### Ethics Statement

This study was conducted in Yixing, China. The impervious-covered and open soils were collected from part of Yixing urban area (31°20′–31°25′N, 119°45′–119°50′E). The selected sampling areas did not involve endangered or protected species, and no specific permissions were required for the soil sampling.

### Study area

The soils in this study were sampled in January 2008 from Yixing City (31°07′–31°37′N, 119°31′–120°03′E), which has an urban area of 13.4 km^2^ in 2000 [3]. Yixing which locates on the plain of the lower reaches of the Yangtze River has had rapid urbanization in recent decades mainly at the expense of agricultural land. Average annual rainfall in Yixing is 1177 mm, and average annual temperature is 15.7°C. The soils in the studied area were formed on the alluvium of the Yangtze River, and the dominant soil type in the agricultural lands around Yixing City is Hydragric Anthrosols [20].

### Soil sampling and analysis

**Figure 1:**
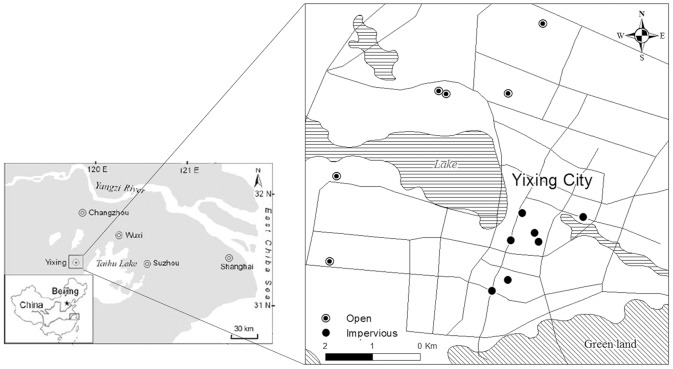
Soil sampling points in Yixing city. Seven sites were selected for impervious-covered soils, and six sites with similar soil parent materials were selected for open soils. Soil samples were collected at 0–20 cm depth.

Seven sites were selected for impervious-covered soils, and six sites with similar soil parent materials were selected for urban open soils (Figure 1). Soil in our study area had SOC content of 1.48 (±0.19 SE, *n* = 9) g kg^−1^, TN content of 1.38 (±0.11 SE, *n* = 9) g kg^−1^, and bulk density of 1.3 (±0.13 SE, *n* = 61) g cm^−3^ in 1982, which these parameters had relatively small variation as their CVs ranged from 8% to 13% (Table 2) [21]. The impervious sites consisted of road pavements, and paved residential squares and alleys, whereas the open area reference sites mainly comprised residential and commercial lawns and gardens, and public greenspaces. Outline description (0–20 cm) for the impervious-covered and open soils selected in our study is shown in Table 3. The soils sampled in our study area primarily had a slit loam texture but varied in the contents of soil admixtures. Compared with the soils in the open areas, the impervious-covered soils had little root penetration and greater amounts of artifacts, including asphalt, cement, brick, tiles, and gypsum. Generally, soil physico-chemical properties were more variable in impervious areas than in the grass areas, and more severely affected by human activities. The mean soil pH and particle fraction larger than 2 mm beneath impervious surfaces (0–20 cm) were 7.72 (range, 7.08–8.33) and 4.28% (range, 6.4%–7.74%), respectively (Table 3).

**Table 2:** Initial properties of the urban soil in Yixing obtained from the second Chinese soil survey ^a^ (*n* = 9 except where noted).

Soil property	0–14 cm		14–25 cm	
	Mean ±SE	CV, %	Mean ±SE	CV, %
Bulk density^ b^, g cm^−3^	1.3±0.13	10	1.44±0.15	10.4
pH	6.6±0.3	4.5	6.9±0.6	8.7
TOC ^c^, %	1.48±0.19	13.3	1.14±0.30	26.4
N, g kg^−1^	1.38±0.11	8.0	1.13±0.28	24.8
C/N	10.6±1.2	11.3	9.9±1.5	15.2
P, %	0.095±0.01	10.5	0.09±0.125	16.7
K, %	1.67±0.09	5.4	1.68±0.11	6.7
Olsen P, mg kg^−1^	5.8±2.8	48.3	5±3.8	76.0
Available K^ d^, mg kg^−1^	85±30	35.3	96±30	31.2
CEC, cmol kg^−1^	19.51±2.36	12.1	20.11±2.02	10.2

a, Office of Soil Survey in Yixing County, conducted in 1979–1982; b, *n* = 61; c, determined using the dichromate oxidation method; d, CH_3_COONH_4_ extractable K.

**Table 3:** Outline description for the urban soils (0–20 cm) selected in our study.

Soil sample	Texture	pH	artifacts, rock fragments, roots	> 2 mm fraction
				%
*Impervious soils*				
1	Silt loam	7.08	coarse angular weathered brick, roots	1.60
2	Silt	7.42	coarse angular freshly weathered rock fragment, roots	9.01
3	Silt loam	7.9	plastic, roots	5.19
4	Silt loam	8.15	coarse angular highly weathered brick and tiles	2.67
5	Silt loam	7.56		8.53
6	Silt	7.58	gypsum, roots	1.44
7	Silt loam	8.33		1.51
*Open soils*				
8	Silt loam	7.21	heavily roots	5.61
9	Silt loam	6.4		0.72
10	Silt loam	7.12	coarse angular weathered rock fragment, roots	4.25
11	Silt loam	7.58	heavily roots	2.78
12	Silt loam	7.74	heavily roots	2.33
13	Silt loam	6.94	heavily roots	2.35

Each sample, consisting of 3 separate soil cores (5 cm in diameter), was taken from 0–20 cm depth, as SOC storage was supposed to be mainly allocated in the upper soil layers. To make the impervious-covered and open soils more comparable, in the impervious areas, only soils having roots penetrations and little mixture of artifacts were selected. We assumed that the selected soil horizon having these properties is of significance in sequestrating SOC. Specific soil horizon from which the soil was sampled was not determined, but the sampled soils had similar soil texture and chemical properties (Table 3). At the impervious sites, we punched holes on the hard ground near the central position of the selected sealing area (2 m×2 m) and removed the padding to make the covered soil accessible for sampling. After collection, soil samples were transported to the laboratory and air dried at room temperature. Then, the air-dried soils were grounded and sieved through a 2-mm nylon mesh; stones, artifacts, and coarse roots greater than 2 mm in size were weighed.

Soil pH was measured in water (1 soil: 2.5 water, w/v) using a glass electrode, and soil particle size analysis was determined using the hydrometer method [22]. Soil organic C concentrations in the samples were determined using the potassium dichromate sulfuric acid oxidation method [23]. Total nitrogen (N) concentrations were measured by Kjeldahl digestion [24]. Soil bulk density was measured by automated three-dimensional laser scanning [25] (NextEngine Desktop 3D Scanner, NextEngine, Inc., US). The densities of SOC and total N in a horizon of unit area (1 m^2^) in each site were calculated as:
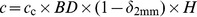
(1)where *c* (kg m^−2^) represents SOC density or total N density, *c*_c_ (g kg^−1^) represents SOC concentration or total N concentration, *BD* (g cm^−3^) represents soil bulk density, *δ*_2mm_ (g g^−1^) represents the fraction of material lager than 2 mm diameter, and *H* (m) represents the soil sampling depth. In our study, the densities of SOC and total N in each site were calculated based on 1 m^2^ square to a 0.2 m depth.

To investigate the stability of SOC in urban areas, a thermostatic soil incubation experiment was conducted. Before the incubation, we first determined soil full water holding capacity. The sieved soil (<2 mm) was packed to the same bulk density (∼1.5 g cm^3^) in cutting ring (100 cm^3^), saturated with water, weighed and then dried at 105°C for 48 hours to determine soil water content. Soil full water holding capacity was the water content of the saturated soil. Fifty grams of each urban soil (<2 mm) were placed into 500-mL capacity plastic bottles and incubated simultaneously in one incubator (SP-300B, Hengyu, China) for 28 days. The soil water content of each sample in the bottle was then adjusted to 60% of full water holding capacity, which the actual amount of water needed was calculated based on the preliminary test. Each plastic bottle was sealed airtight and incubated in the dark at 25°C. Each sample was pre-incubated without CO_2_ absorption for 5 days [18]. Then, a beaker with 5 mL of 0.6 mol L^−1^ NaOH was placed in each jar to absorb the evolved CO_2_ during the incubation. Control jars (without soil) were used to measure the background CO_2_ concentration. The NaOH was renewed after 1, 3, 5, 7, 14, 21, and 28 days. Any loss of water from the cylinders (checked by weighing) was corrected by a mist sprayer. The CO_2_ trapped in the NaOH was determined by back-titration of excess NaOH with 1.5 mol L^−1^ H_2_SO_4_ after precipitation with 1 mol L^−1^ BaCl_2_ [26].

A first-order decay equation was used to describe the organic C mineralization in the samples studied [27]:
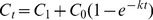
(2)where *C_t_* (mg C g^−1^ C) is the cumulative amount of SOC mineralized during time t (day), *C*_1_ (mg C g^−1^ C) is the rapidly mineralizable SOC pool, *C*_0_ (mg C g^−1^ C) is the potentially mineralizable SOC pool, and *k* (mg C g^−1^ C d^−1^) is the corresponding mineralization rate constant.

The SOC mineralization half-time (i.e., time required to mineralize half of the potentially mineralizable SOC) was calculated as follows [28]:
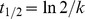
(3)


### Statistical analysis

Model fit was conducted using the Global curve fit wizard in SigmaPlot 12.0 software package (Systat Software, Inc., Chicago, IL, USA). Linear regression analysis and the comparisons in the densities of SOC and total N and the model parameters between the impervious and open land uses (group t-test) were conducted using SAS 8.2 software (SAS Institute, Cary, NC).

## Results and Discussion

### SOC density under impervious surfaces

**Figure 2:**
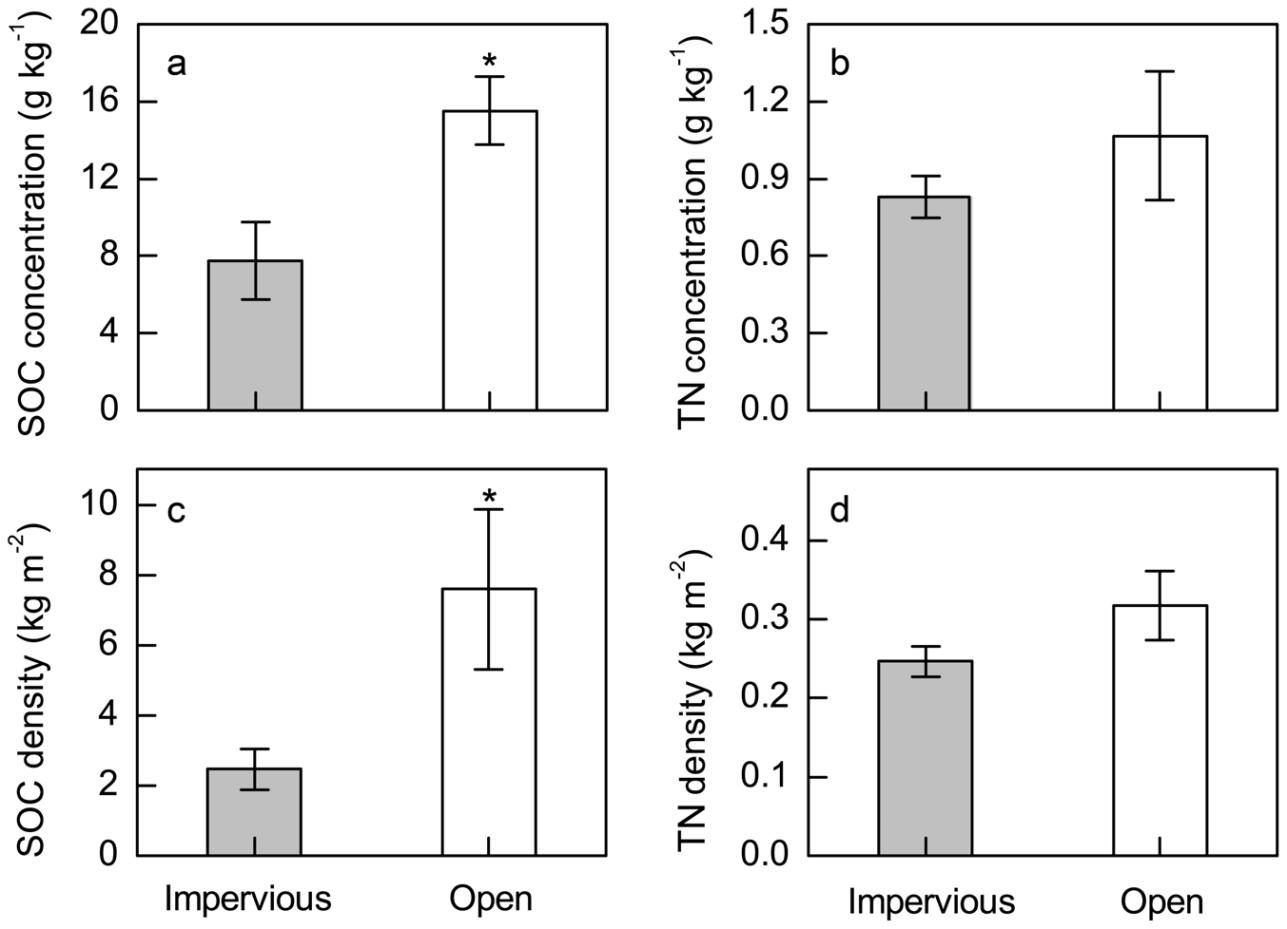
The concentrations and densities of SOC and TN for the impervious-covered and open soils in Yixing city. Values are means ± SE, SOC represents soil organic carbon, and TN represents total nitrogen, * *P*<0.05 (*n* = 7 for urban impervious-covered soils, and *n* = 6 for urban open soils).

The impervious-covered sites had lower SOC and TN concentrations (0–20 cm) compared with the open sites and those in 1982 (Figure 2a, b). The SOC and TN concentrations of the open soils were comparable with those in 1982. The mean SOC density beneath impervious surfaces was 68% (±7.7% SE, *P*<0.05) lower than that in open areas (2.46 versus 7.59 kg m^−2^, respectively, Figure 2c). Similar results were also reported by Pouyat *et al*. (2006) and Raciti *et al*. (2012) that SOC densities under impermeable surfaces were lower than that of open soils [8], [15]. It is interesting to note that the SOC densities observed at the impervious sites were comparable to those in New York City (Raciti *et al*. 2012) although the samples were distributed at different cities [15], suggesting that there might be an equilibrium value for the depletion of SOC densities under impervious surfaces. The total N densities were 0.25 kg m^−2^ and 0.32 kg m^−2^, respectively, for the impervious-covered and open soils in Yixing City, but no significant difference was found between them (Figure 2d), probably due to the relatively small sample size in our study.

The exact mechanisms of SOC loss from the impervious areas are unknown, but likely possibilities include gaseous losses, aqueous losses as dissolved organic and inorganic C, and physical removal of topsoil during the construction process. The removal of surface soil may readily result in a significant depletion of organic C stock due to enhanced mineralization and re-use. The situation could be worse when topsoil is not re-used and is left slowly to decompose. If the potential loss of SOC inferred by this study hold true for other impervious-covered soils, this would suggest that roughly 0.45 Pg (1 Pg = 10^15^ g) of SOC would be potentially lost in China associated with 87128 km^2^ of impervious surface [6]. The potential loss of SOC stock could be more significant in magnitude when taking into account the large vegetable C losses in response to initial land use change to urban.

Although the SOC densities beneath impermeable surfaces were relatively lower, it is not adequate to exclude these C pools for estimates of ecosystem C stocks in national scale as required by Kyoto Protocol signatories [10]. In fact, investigation of SOC stocks and dynamics in urban impervious areas are getting more important to the C study, since there is ongoing significant expanding of constructed impervious surfaces around the world [5], [6]. In our study, soil samples were collected at 0–20 cm soil layer and therefore SOC densities were calculated to 0.2 m depth. Urban subsoil horizons may also contain considerable amounts of SOC since physical disturbance (e.g., mixing, burying) can result in translocation of topsoil that is rich in OC to deeper soil layer [29]. Thus, the SOC storage underlying capped surfaces in urban areas could be larger than we estimated when it was calculated to 1 m soil depth as usually did. The SOC inventory involving subsoil horizons beneath impervious surfaces in urban areas will strengthen our understanding on the impact of urbanization on the ecosystem. Furthermore, the SOC inventory in urban area should consider the definition and built-up age of the urban used by the investigators. Urban areas, regardless of definition, are rapidly expanding at unprecedented rates; inconsistent definitions of ‘urban’ will result in different conclusions about the size of urban C stocks [30]. Moreover, it was suggested that the built-up age could modify urban C stocks [31].

**Figure 3:**
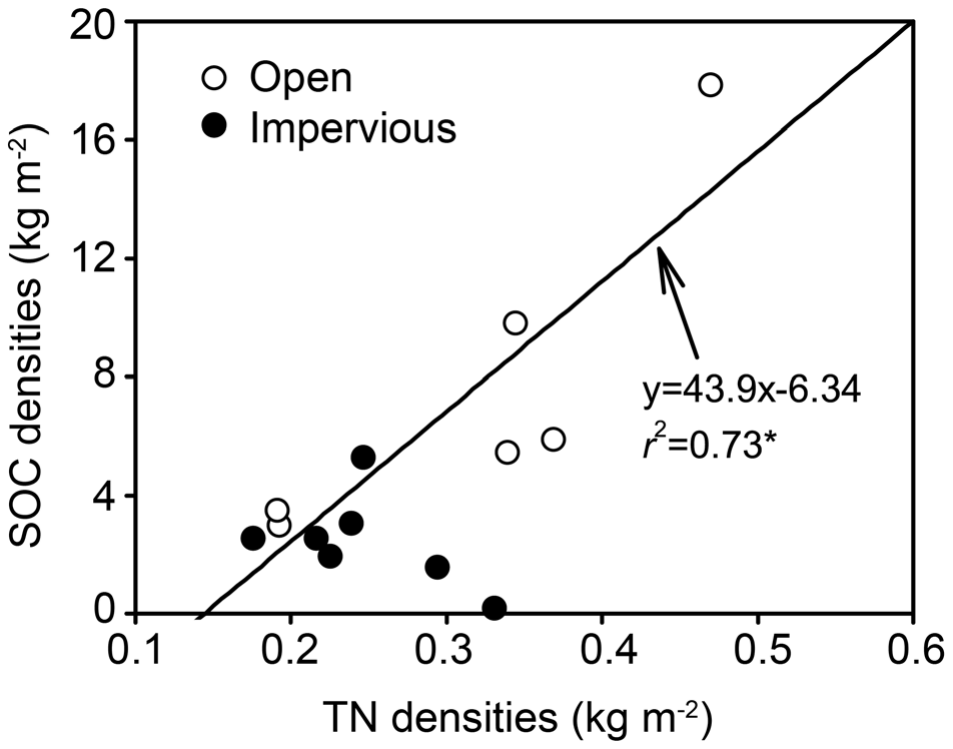
The correlations between the densities of SOC and TN for urban soils in Yixing city. SOC represents soil organic carbon, and TN represents total nitrogen, * *P*<0.05 (*n* = 7 for urban impervious-covered soils, and *n* = 6 for urban open soils).

In most soils, there is a tight coupling between the stocks and fluxes of C and N. The mean C/N ratio in the soils covered by impervious surfaces was significantly lower than the ratio in open soils (10.8 versus 22.1, respectively, *P*<0.05, data not shown), indicating that the microorganisms had a relative C deficit state in the impervious-covered soils. The higher C/N ratios in the open soils were probably due to the abundant input of organic materials (e.g., leaves, branches) from plant as these materials always had relatively higher C/N ratios [32]. Regression of SOC density as a function of total N density in the open soils revealed a strong linear relationship between the two variables at the 0–20 cm depth (*r* ^2^ = 0.73, *P*<0.05, Figure 3). In contrast, there was no clear relationship between SOC and total N for the soils beneath impervious surfaces, suggesting that the paving in urban areas decoupled the cycle of C and N in the soils [15].

Installation of impervious surfaces in urban areas had negative impact on urban ecosystem, which was indicated by large amount of SOC loss that can offset the C stored in trees or other green vegetation in urban areas, and perturbed soil C and N cycling in impervious area. More greenspaces or semi-pervious pavement systems were, therefore, recommended in future urban construction to mitigate the negative consequences of urban artificial soil sealing [5]. In addition, soil management (e.g., fertilization, tillage, irrigation) for the open sites could be optimized to enhance the SOC sequestration in urban soils.

### SOC stability under impervious surfaces

**Figure 4:**
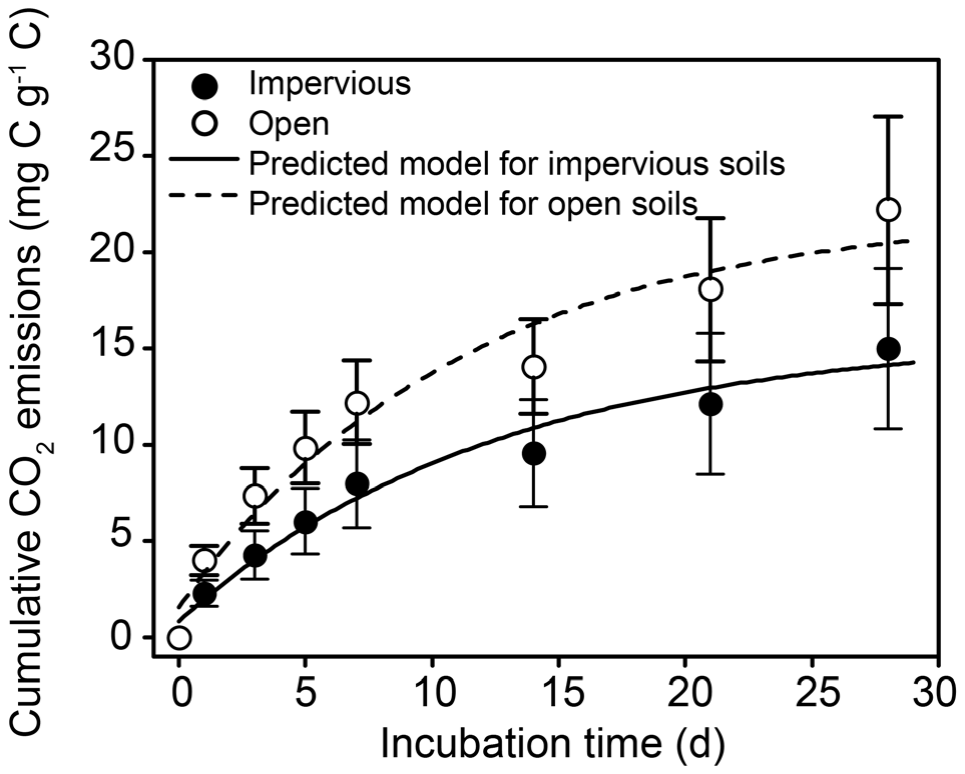
Cumulative carbon release (as CO_2_) from the impervious-covered and open soils during the 28-d incubation. Data were fitted by the first-order decay model. The bars indicate standard errors (*n* = 7 for urban impervious-covered soils, and *n* = 6 for urban open soils).

The laboratory incubation can provide an insight of SOC dynamics (e.g., [33]). The amount of SOC mineralized (mg C g^−1^ C) over the 28-d incubation was calculated based on the total (whole soil) SOC content in each sample to enable us directly compare the potential C loss (i.e., CO_2_ emissions) from the impervious-covered and open soils, because the portion of SOC is comparable amongst urban soils although the initial SOC content for the urban soils were unequal (Figure 4). It was found that less CO_2_ was emitted from the soils underlying capped surfaces than from the open soils during the incubation, indicating that the SOC was more stable beneath impervious surfaces than in open areas. The lower transformation of organic C in the covered soils can be partially explained by a low microbial activity in these soils [34].

The first-order decay model (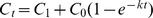) used in this study fitted the data well (*r* ^2^ were 0.97 and 0.96, Table 4). The easily decomposable SOC pool (*C*_1_) was small relative to the potentially decomposable SOC pool (*C*_0_) in all samples. The *C*_1_ and *C*_0_ of SOC under impervious surfaces were 47% and 27%, respectively, lower than those in open areas, indicating that there is a more severe depletion of readily decomposable SOC pool after paving. The impervious-covered and open soils had similar mineralization rate constants (*k*) of the potentially decomposable organic C. However, the lower value of *C*_0_ *k*, a parameter that could be comparable with the initial potential rate of SOC mineralization [35], together with the longer mineralization half-life time (*t*_1/2_) of SOC under impervious surfaces revealed these soils had weaker organic C decomposability and lower turnover rate.

**Table 4:** The first-order decay model (Eq. 2) parameters and coefficients of determination (*r* ^2^) for carbon mineralization in urban impervious-covered and open soils.

Parameters	Impervious	Open	
*C*_1_ (mg C g^−1^ C)	0.84	1.59	n.s.
*C*_0_ (mg C g^−1^ C)	14.9	20.53	n.s.
*k* (mg C g^−1^ C d^−1^)	0.08	0.09	n.s.
*C*_0_ *k*	1.17	1.87	n.s.
*t*_1/2_ (d)	8.84	7.62	n.s.
*r* ^2^	0.97	0.96	n.s.
*p*	0.01	0.01	n.s.

*C*_1_, rapidly mineralizable SOC pool; *C*_0_, potentially mineralizable SOC pool; *k*, SOC mineralization rate constant; *C*_0_ *k*, a parameter that could be comparable with the initial potential rate of SOC mineralization [35]; *t*_1/2_, SOC mineralization half-time.

n.s., not significant.

Edmondson *et al*. (2012) suggested that the turnover of SOC beneath impervious surface likely depend upon the type of capping and the extent of impervious surface [10]. In our study, the covered soils were collected conformably from areas with more than 95% impervious surface, in which the exchanges of gas and water between the soil and atmosphere were supposed to be rare. In fact, there are some urban impervious-covered soils (e.g., patio, garden path) which were distributed in areas dominated by vegetation. The soils underlying capped surfaces in these areas could be colonized by the root systems of lawn grasses and garden trees and shrubs. Thus, it is likely that below these smaller patches of impervious surfaces the soil remains active potentially accumulating SOC and has more robust organic C transformation. Accordingly, more systematic research and soil samples including different land use types and sealing degrees in urban areas are required to better understand the SOC turnover under impervious surfaces.

Additionally, some impervious surfaces in urban area may be removed due to urban land use change. The differences in soil biochemical properties between the impervious and open sites are supposed to be minimized, since the removal of impervious surface. Investigation on the temporal dynamic of SOC after removal of sealing will be beneficial for comprehensively understanding the ecological effects of urbanization. Therefore, management and time, which were needed to rebuild organic C sequestration capacity for the soil after removal of sealing, need to be studied.

## Conclusions

Our data demonstrated that the SOC density decreased at 0–20 cm depth in urban areas (regardless of the definition) after the installation of impervious surfaces, although the precise mechanism of organic C loss from these soils was uncertain. The artificial soil sealing in urban areas also decoupled the cycle of C and N, since SOC density correlated positively with total N density in the open soils (*P*<0.05), but they did not exhibit an apparent relation in the impervious-covered soils. The SOC underlying capped surfaces had weaker decomposability and lower turnover rate compared with that in open areas, which was indicated by a smaller readily decomposable SOC pool, a longer half-time, and a smaller amount of CO_2_-C emission during the 28-d incubation. More greenspaces or semi-pervious pavement systems in future urban construction will mitigate the negative consequences of urban artificial soil sealing.
